# Two-Month Individually Supervised Exercise Therapy Improves Walking Speed, Step Length, and Temporal Gait Symmetry in Chronic Stroke Patients: A before–after Trial

**DOI:** 10.3390/healthcare10030527

**Published:** 2022-03-14

**Authors:** Kiyoshi Yoshioka, Tatsunori Watanabe, Norikazu Maruyama, Mizuki Yoshioka, Keita Iino, Kimikazu Honda, Koshiro Hayashida

**Affiliations:** 1 Kumamoto Center, Rehabilitation Center for all Customers with Stroke and Cerebrovascular Diseases, SENSTYLE Inc., Kumamoto 860-0088, Japan; nanntecotta.mzk@gmail.com (M.Y.); pt014kt0823ay0623@gmail.com (K.I.); kimikazu.h@kcr.ac.jp (K.H.); rihasen.kumamoto@gmail.com (K.H.); 2 Department of Muscle Development and Regeneration, Division of Organogenesis, Institute of Molecular Embryology and Genetics, Kumamoto University, Kumamoto 860-0811, Japan; 3 Department of Sensorimotor Neuroscience, Graduate School of Biomedical and Health Sciences, Hiroshima University, Hiroshima 734-8553, Japan; twatan@hiroshima-u.ac.jp; 4 Department of Physical Therapy, Faculty of Fukuoka Medical Technology, Teikyo University, Fukuoka 836-8505, Japan; maru@fmt.teikyo-u.ac.jp; 5 SENSTYLE Institute for the Science of Aging, Kumamoto 860-0088, Japan

**Keywords:** stroke, hemiparesis, gait asymmetry, exercise therapy, chronic phase

## Abstract

Gait asymmetry is common after stroke and is a major risk factor for falls. In particular, temporal gait asymmetry often remains in the chronic stage of stroke. However, health insurance does not cover rehabilitation for patients with chronic stroke in many countries. Accordingly, it is undetermined whether individually supervised exercise therapy has beneficial effects on chronic hemiparetic gait. Patients with stroke (*n* = 25) more than 6 months after onset performed 70 min of individually supervised exercise twice weekly for 2 months in 16 sessions with qualified personnel. The intervention significantly reduced the pre-swing phase on the paretic side (mean = 91.8%, 95%CI, 84.8–98.8). In addition, there was a significant improvement in pre-swing phase symmetry in those with great asymmetry prior to the intervention (*p* = 0.022). Step length significantly increased after the intervention on both sides (non-paretic, *p* = 0.029; paretic, *p* = 0.0055). Walking time at both comfortable and maximum speeds was significantly shortened (comfortable, *p* = 0.0041; maximum, *p* < 0.0001). Our findings suggest that there remains scope to improve gait ability with individually supervised exercise therapy in patients with chronic stroke, whose functional recovery is often considered unlikely. This type of intervention may be a simple and effective option to improve gait parameters, including temporal asymmetry, even in patients with chronic stroke.

## 1. Introduction

Stroke is a leading cause of disability worldwide [1], and functional walking ability is an important outcome for patients following stroke. Many patients continue to have residual gait deficits and have difficulty returning to community living and their life roles in the chronic post-stroke stage [2,3]. Hemiparetic gait patterns are typically characterized by spatiotemporal asymmetry [4,5], which is related to reduced walking efficiency [6] and is a significant risk factor for falls in patients following stroke [7,8]. Thus, gait asymmetry is commonly targeted in post-stroke rehabilitation to facilitate the recovery of walking function [9,10].

Treadmills are widely used in rehabilitation facilities. Repeated rehabilitation using either a normal or split-belt treadmill improves walking speed and step length asymmetry in patients with chronic stroke [9,10]. However, evidence shows that neither a treadmill nor a split-belt treadmill training improves temporal asymmetry (e.g., stance and pre-swing (double-support) phases) [10,11]. In addition, orthoses have long been used to improve walking ability in patients following stroke. In particular, ankle-foot orthoses improve the asymmetry of stance time of the gait cycle [12] but not dynamic balance [13]. Furthermore, in the past several decades, unique and novel rehabilitation interventions have been developed to meet the increasing need for patients to recover further in the post-stroke chronic stage. For example, robot-assisted gait training [14] and wearable hybrid assistive limb devices to assist walking movements [15] improve walking ability and associated gait parameters, including asymmetry.

In Japan, as well as in some other countries, rehabilitation costs are not covered by health insurance plans 6 months after stroke onset (chronic stage); thus, hospitals and clinics do not provide rehabilitation for patients in the chronic stroke stage, implying that there is no standard rehabilitation for these patients [16]. Although geriatric health services are available to elderly people, including those with hemiparesis in the chronic stage of stroke, exercise programs are typically provided only to maintain general health [17,18]. In addition, there are no programs available for younger working-age patients who wish to continue rehabilitation in the post-stroke chronic stage in order to return to work. In the last decade, self-pay rehabilitation has become available to meet the needs of patients who, following stroke, desire to continue their rehabilitation in the chronic stage. At facilities providing self-pay rehabilitation, individually supervised exercise therapy is provided by qualified personnel, such as physical and occupational therapists. However, the effectiveness of this type of intervention on physical performance has not yet been reported. Considering previous reports indicating the importance of lower-leg strength in symmetric gait [19,20], as well as the improvement of asymmetric gait in patients with chronic stroke through the use of visual and proprioceptive feedback [9], we hypothesized that individually supervised exercise therapy focusing on these aspects has a beneficial effect on chronic hemiparetic gait. Specifically, a combination of individualized strength training and locomotor exercises with appropriate feedback supervised by qualified personnel has the potential to improve gait parameters, including both spatial and temporal asymmetry, in patients with chronic stroke. Accordingly, the purpose of this study was to investigate the effect of individually supervised exercise therapy provided at a self-pay rehabilitation facility on gait parameters in patients with chronic stroke.

## 2. Materials and Methods

### 2.1. Design and Subjects

**Figure 1:**
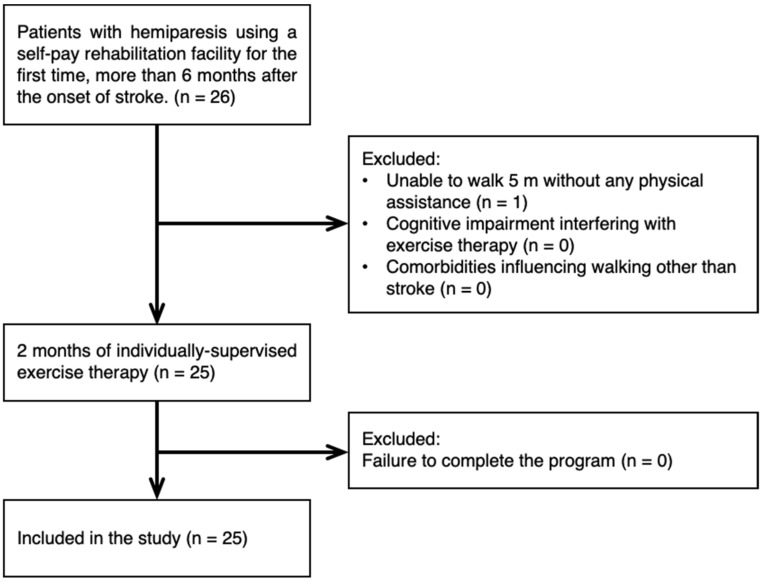
Flow chart of inclusion and exclusion process.

This was a single-center, quasi-experimental before–after trial. Using a convenient sampling, we recruited participants from first-time users of a self-pay rehabilitation facility more than 6 months after the onset of stroke (Figure 1). The median period from onset was 19 months (range: 6 to 192). The inclusion criteria were the presence of hemiparesis and ability to walk 5 m without physical assistance. The exclusion criteria were the presence of cognitive impairment interfering with exercise therapy and comorbidities other than stroke influencing walking. The characteristics of the subjects included in this study are presented in Table 1. 

### 2.2. Intervention

A series of intervention sessions was provided individually to each subject. Each session was 70 min in duration and included 50 min of exercise therapy supervised by a physical therapist and 20 min of strength training supervised by a physical therapist/athletic trainer. Hereafter, these two types of interventions will be collectively described as exercise therapy. The content of the exercise therapy varied according to each subject’s condition and ability and was adjusted by the assigned therapist. The most common intervention types were treadmill and ergometer exercises, with appropriate feedback from the therapist as needed. Depending on the patient’s physical level, body weight or free weight training was used, mainly to train the lower-limb and trunk muscles. The details of the exercise program are provided in online supplement (DOI: 10.17632/84g8yxw5gh.1). The intensity, duration, and frequency of each exercise were determined by the therapist/athletic trainer responsible for the session, with reference to a heart rate monitor (OH1, POLAR). The intensity of exercise was within 80% of the maximum heart rate [21] (approximately 125 bpm in the subjects) and was determined by the therapist in charge according to the patient’s condition. The intervention session was provided twice a week for 8 weeks (a total of 16 sessions). Gait parameters were evaluated before and after the series of intervention sessions.

### 2.3. Measurement and Analysis

The subjects performed two types of walking: comfortable walking with the instruction, “Please walk comfortably at your usual walking speed,” and maximum-speed walking with the instruction, “Please walk as quickly as possible”. A therapist walked close alongside some subjects who were judged to require proximal supervision for fall prevention, but s/he did not guide or instruct the walking during the recording.

Comfortable-speed walking was captured at 60 frames per second with a camera (P1365 MkII, AXIS) fixed at a height of 150 cm on the side of the walkway. Marking tapes were placed at 1 m intervals in the walkway and used for calibration of the video-captured images. The acquired data were analyzed using Kinovea (version 0.8.15, Kinovea), and the following parameters were calculated: the ratio of the stance and swing phases in a gait cycle, walking speed, walking pitch, and step length. Specifically, the video stream was divided into frames using Kinovea, and the frames with the ground contact and foot release were identified to obtain their timings. A gait cycle was defined as the time from initial ground contact of one foot to subsequent ground contact of the same foot [22], and the percentage of each gait phase was computed. The definitions of gait phases followed Perry’s (Rancho Los Amigos) observational gait assessment [23]. Briefly, the period from the initial contact of the foot with the ground until immediately before it departed from the ground was defined as the stance phase, and the period during which the plantar surface of the foot was not in contact with the ground was defined as the swing phase. In addition, the period during which both feet were in contact with the ground immediately before the swing of the rear foot was defined as the pre-swing phase. Accordingly, one gait cycle was divided into 4 periods (i.e., swing and pre-swing phases for the paretic and non-paretic sides). Step length was identified by marking the heel position during the ground contact on the Kinovea analysis screen. Walking speed was defined as the distance the acromion moved during two gait cycles divided by the time of two gait cycles. We also calculated asymmetry for three gait parameters: stride length, swing phase, and pre-swing phase (asymmetry = paretic/non-paretic). The time to walk 5 m was assessed during maximum-speed walking, and we adopted the shortest duration of two trials.

### 2.4. Statistical Analysis

Prior to the comparison of the parameters before and after the intervention, the Shapiro–Wilk test was used to verify the normal distribution of the data. For normally distributed parameters, *p* values were calculated using a two-tailed paired Student’s t-test, whereas non-normal variables were tested using the Wilcoxon signed-rank test. Effect size, *r*, was calculated using formula *r* = $\sqrt{\frac{t^{2}}{t^{2}+df}}$, for the paired samples *t*-tests and using formula *r* = $\frac{Z}{\sqrt{n}}$ for the Wilcoxon signed-rank tests. Effect size statistics, *r*, were assessed against Cohen’s criteria (0.1 = small, 0.3 = medium, 0.5 = large effect) [24]. For all statistical tests, *p* < 0.05 was regarded as statistically significant. Statistical tests were performed with R software (version 4.0.2) or Prism 9 (version 9.3.1; GraphPad, San Diego, CA, USA).

### 2.5. Ethical Considerations

Prior to the experiment, the subjects were fully informed about the study in writing and orally. Their consent was also obtained with a signature (subjects themselves or their family members if they had difficulty writing). This study was conducted in accordance with the Declaration of Helsinki and with an approval of the ethics committee of the institution (approval number: 19-003). The study protocol was registered in the UMIN Clinical Trial Registry (UMIN000043620)

## 3. Results

**Figure 2:**
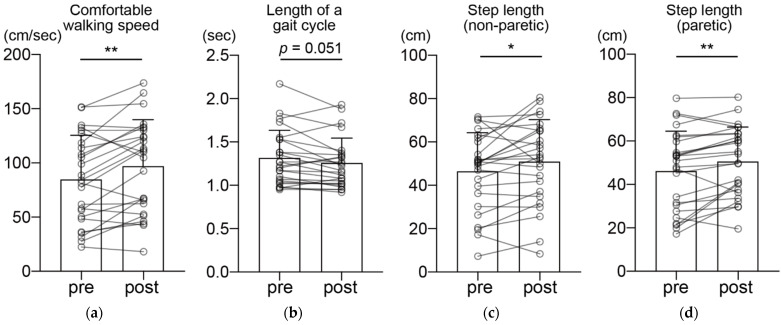
Pre- and post-intervention comparison of gait parameters during comfortable walking speed: (**a**) comfortable walking speed, (**b**) length of a gait cycle, (**c**) non-paretic step length, and (**d**) paretic step length. The connected plots show the pre- and post-intervention data of a subject. Bar graphs show the mean value. * *p* < 0.05, ** *p* < 0.01, error bar = SD, *n* = 25.

All 25 participants who met the initial inclusion criteria completed the 16 training sessions in 2 months. No adverse events were reported during the intervention period. Comfortable walking speed significantly increased after the intervention (Figure 2a; pre, 85.2 ± 40.1 cm/s; post, 97.6 ± 42.9 cm/s, *p* = 0.0041, *t* = 3.176, *r* = 0.54). There was no significant change in the length of a gait cycle, although there was a decreasing trend after the intervention (Figure 2b; pre, 1.31 ± 0.32 s; post, 1.25 ± 0.29 s, *p* = 0.051, *Z* = 2.90, *r* = 0.52). Step length was significantly elongated after the intervention on both the non-paretic and paretic sides (Figure 2c; non-paretic side, pre, 46.6 ± 18.4 cm; post, 50.9 ± 16.0 cm, *p* = 0.029, *t* = 2.317, *r* = 0.43; Figure 2d; paretic side, pre, 46.4 ± 17.9 cm; post, 50.9 ± 19.4 cm, *p* = 0.0055, *t* = 3.053, *r* = 0.43).

**Figure 3:**
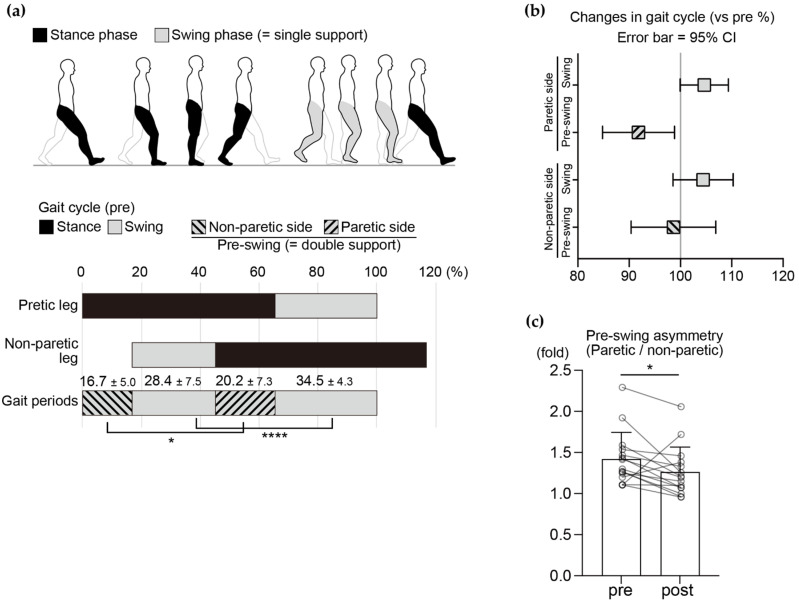
Proportion of gait cycle prior to the intervention and changes in gait cycle after the intervention. (**a**) Illustration shows the gait cycle (stance phase and swing phase). The pre-swing phase (double support phase) is the time spent with both feet in contact with the floor. The graph shows the pre-intervention gait cycle (mean ± SD, * *p* < 0.05, **** *p* < 0.0001, *n* = 25). (**b**) Percent change in gait phase after the intervention. * *p* < 0.05, n.s. not significant; Error bar = 95%; confidence interval, *n* = 25. (**c**) Fold change in pre-swing asymmetry (paretic/non-paretic) in a subgroup with >1.1-fold asymmetry at the baseline (pre-intervention). The connected plots show the pre- and post-intervention data of a subject. Bar graphs show the mean value. * *p* < 0.05, error bar = SD, *n* = 15.

There was no significant change in stride-length symmetry (paretic/non-paretic; pre, 1.07 ± 0.34, post, 1.11 ± 0.40, *p* > 0.99). Temporal asymmetry in the gait cycle, a well-known feature in patients with hemiparesis, was observed in the patients in this study (Figure 3a). Although there was no significant change in the swing-phase symmetry after the intervention (paretic/non-paretic; pre, 1.09 ± 0.08; post, 1.11 ± 0.10, *p* = 0.38, *Z* = 0.053, *r* = 0.011), there was an improving trend in the pre-swing phase symmetry (paretic/non-paretic; pre, 1.23 ± 0.35; post, 1.15 ± 0.33, *p* = 0.089, *Z* = 1.69, *r* = 0.34). This improvement may be explained by a significant decrease in the proportion of the pre-swing phase on the paretic side (Figure 3b, post-intervention changes in each phase of the gait cycle (vs. pre %); non-paretic pre-swing phase, mean = 98.6%, 95%; confidence interval, 90.4 to 106.9; non-paretic swing phase, mean = 104.4%, 95% CI, 98.6 to 110.3; paretic pre-swing phase, mean = 91.8%, 95% CI, 84.8 to 98.8; paretic swing phase, mean = 104.7%, 95% CI, 100.0 to 109.3). As some previous studies have demonstrated, improvements in gait symmetry depend on the initial gait asymmetry level [25,26]; therefore, we divided the subjects into two subgroups depending on the pre-swing phase asymmetry level at baseline (>1.1-fold in the paretic/non-paretic pre-swing ratio, *n* = 15) [27]. Statistical analysis revealed that pre-swing phase asymmetry improved significantly in the subgroup with greater asymmetry at baseline (Figure 3c, pre, 1.42 ± 0.33; post, 1.26 ± 0.30, *p* = 0.022, *Z* = 2.30, *r* = 0.46).

**Figure 4:**
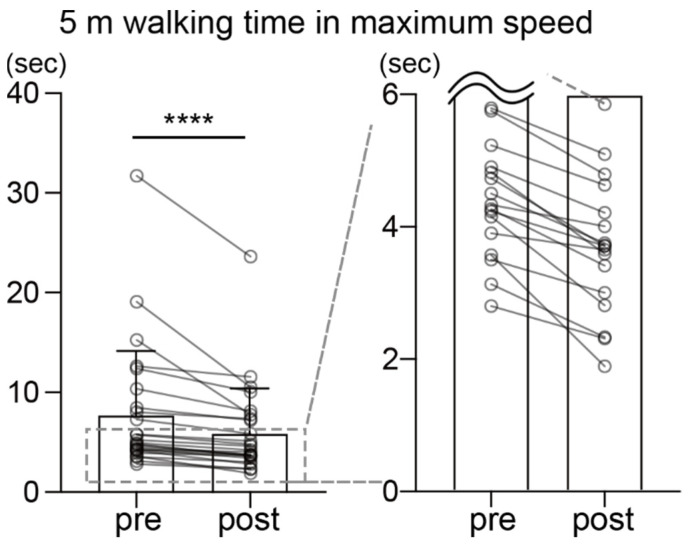
Pre-/post-comparison of 5 m maximum-speed walking time. Each plot shows the data of a subject, and the pre- and post-intervention data are connected by lines. The bar graph shows the mean value. The graph on the right is a magnified view of the area enclosed by the dotted line. **** *p* < 0.0001, error bar = SD, *n* = 25.

Finally, the time to walk 5 m at the maximum speed was significantly reduced after the intervention (Figure 4; pre, 7.79 ± 6.48 s; post, 5.96 ± 4.55 s, *p* < 0.0001, *Z* = 4.37, *r* = 0.87).

## 4. Discussion

In this study, we investigated whether individually supervised exercise therapy has beneficial effects on gait parameters in patients with chronic stroke. To the best of our knowledge, this is the first study to demonstrate improvements in gait parameters, including temporal gait symmetry, after 2 months of individually supervised exercise therapy in patients with chronic stroke.

Each phase of the gait cycle was used as an objective indicator to evaluate the overall gait quality. In the gait of healthy individuals, the swing phase of each limb is approximately 40%, and the pre-swing phase of each limb is approximately 10% [28]. Meanwhile, the gait of patients with hemiparesis is characterized by a decrease in the proportion of the swing phase [29,30] and by left–right asymmetry, with a prolonged pre-swing phase on the paretic side and a shortened swing phase on the non-paretic side [27,31]. In the present study, we confirmed that our subjects also showed a typical hemiparetic gait cycle at baseline characterized by asymmetry and a prolonged pre-swing phase by two-fold on the paretic side compared to those in healthy individuals as previously reported [28]. Gait asymmetry leads to lower walking energy cost and balance [8] and is a predictor of falls with high sensitivity [7]. In the present study, after the individually supervised exercise therapy, the proportion of the pre-swing phase on the paretic side decreased, and the temporal symmetry of the pre-swing phase improved, especially in patients who showed greater asymmetry at baseline. It is possible that feedback from therapists during locomotor training may have had beneficial effects on these gait parameters because external feedback improves gait symmetry [9,32]. As the pre-swing phase is a period of load transfer from the hindfoot to the forefoot, indicating that the pre-swing phase on the paretic side in patients following stroke is a preparation period for swinging of the paretic side [33], improvements in step length may have been driven by improvements in the pre-swing phase parameters. Although improving temporal gait symmetry is a difficult clinical challenge, particularly in the chronic stage [34], our findings indicate that individually supervised exercise therapy may be a way to overcome this challenge. Overall, this type of intervention may improve the gait cycle in patients with chronic stroke.

Walking speed is a good indicator of gait independence. A decrease in walking speed is related to reduced community participation and quality of life in stroke survivors [35]. In the present study, a significant increase in comfortable walking speed was observed after the intervention. In addition, a significant increase in step length and tendency toward a shorter gait cycle were observed. As a shorter step length and elongated gait cycle are the main causes of reduced walking ability in patients following stroke [36], the changes in these parameters observed in the present study are indicative of an improvement in the subjects’ walking ability. Reflecting the improvement in gait parameters during comfortable-speed walking, the 5 m maximum-speed walking time improved in all 25 subjects. Together with the gait-cycle results, the walking ability of patients with hemiparesis significantly improved after 2 months of individually supervised exercise therapy.

There are several limitations that should be acknowledged in this study. First, this study was quasi-experimental without control; thus, we cannot completely rule out the possibility that the functional improvements were driven by natural recovery, although all the patients in this study were considered “chronic” in the health insurance system. Further studies are necessary to reach concrete conclusions. Secondly, this study was conducted in a single center in Japan with a relatively small sample size and a wide range of time post-stroke among participants. Post hoc power analysis (using G*Power, version 3.1.9) revealed a power of between 0.62 and 0.96, with an alpha level of 0.05; the lower power suggests a possibility of type II error in our data. Therefore, the generalizability to a larger/particular population and to the other countries is not clear. Third, this study lacks information about the lesion location, size, and degree of paralysis at onset. Access to or sharing of patients’ medical information among facilities is a future challenge for self-pay rehabilitation facilities. Importantly, however, although a wide variety of patients with chronic stroke were included in the present study, maximum-speed walking time improved in all the subjects. This indicates that there is scope for improvement, even in the chronic stage.

## 5. Conclusions

A significant implication drawn from this quasi-experimental study is that walking ability, particularly temporal gait symmetry, may be improved by individually supervised exercise therapy in patients following stroke, even in the chronic stage, during which the cost of rehabilitation is not covered by health insurance plans. Along with other rehabilitation protocols that improve gait parameters and ability in patients following stroke, individually supervised exercise therapy, which does not require expensive devices and technologies, may be an option for patients who wish to continue recovering their functional skills in the chronic stage.

## Figures and Tables

**Table 1:** Demographic data of the subjects.

Characteristics	
*n*	25
Sex (male/female)	18/7
Age (years)	61.9 ± 11.1
Type of stroke (infraction/hemorrhage)	13/12
Paretic side (left/right)	18/7
Median period from onset (months)	19 (range: 6 to 192)

Data are shown as mean ± standard deviation. Period from the onset indicates months elapsed to the initial measurement.

## Data Availability

The data that support the findings of this study are openly available in Mendeley Data. DOI: 10.17632/84g8yxw5gh.1.
